# Risk of Obstructive Sleep Apnea with Daytime Sleepiness Is Associated with Liver Damage in Non-Morbidly Obese Patients with Nonalcoholic Fatty Liver Disease

**DOI:** 10.1371/journal.pone.0096349

**Published:** 2014-04-24

**Authors:** Edoardo Alessandro Pulixi, Eleonora Tobaldini, Pier Maria Battezzati, Paola D'Ingianna, Vittorio Borroni, Anna Ludovica Fracanzani, Marco Maggioni, Serena Pelusi, Mara Bulgheroni, Massimo Zuin, Silvia Fargion, Nicola Montano, Luca Valenti

**Affiliations:** 1 Department of Pathophysiology and Transplantation, Università degli Studi di Milano, Internal Medicine, Fondazione IRCCS Ca' Granda Ospedale Policlinico di Milano, Milano, Italy; 2 Department of Biomedical and Clinical Sciences “L. Sacco”, Division of Medicine and Pathophysiology, Università degli Studi di Milano, Ospedale Luigi Sacco, Milano, Italy; 3 Department of Health Sciences, Università degli Studi di Milano, Division of Medicine and Gastroenterology and Liver Unit, Ospedale San Paolo, Milano, Italy; 4 Pathology, Fondazione IRCCS Ca' Granda Ospedale Policlinico di Milano, Milano, Italy; 5 International Clinical Research Center, St. Anne University Hospital, Brno, Czech Republic; Copenhagen University Hospital Gentofte, Denmark

## Abstract

**Background:**

A high prevalence of obstructive sleep apnea syndrome (OSAS) has been reported in severely obese patients with nonalcoholic fatty liver disease (NAFLD), but few studies have evaluated OSAS in non-morbidly obese NAFLD patients.

**Aims:**

To determine the prevalence of risk for OSAS with or without daytime sleepiness in non-morbidly obese patients with NAFLD and evaluate the association with the severity of liver damage.

**Methods:**

We considered 159 consecutive patients with histological NAFLD and body mass index (BMI) <35 Kg/m^2^, and 80 controls without ultrasonographic steatosis matched for age, sex, and BMI. OSAS risk was determined by positivity for Berlin questionnaire (BQ), and daytime sleepiness by the Sleepness Epworth Scale (ESS). Liver damage was evaluated according to the NAFLD activity score.

**Results:**

In NAFLD patients, BQ alone was positive in 39 (25%), ESS in 8 (5%), and both in 13 (8%, OSAS with sleepines); p = ns vs. controls without steatosis. In NAFLD patients at risk for OSAS with (but not in those without) sleepiness, we observed a higher prevalence of nonalcoholic steatohepatitis (NASH; 11/13, 85% vs. 72/146, 49%; p = 0.018), and of clinically significant fibrosis (stage>1; 9/13, 69% vs. 39/146, 27%; p = 0.003). At multivariate logistic regression analysis, OSAS with sleepiness was strongly associated with NASH and fibrosis>1 independently of known clinical risk factors such as age, gender, BMI, diabetes, and ALT levels (OR 7.1, 95% c.i. 1.7–51, p = 0.005 and OR 14.0, 95% c.i. 3.5–70, p = 0.0002, respectively).

**Conclusions:**

A proportion of NAFLD patients without severe obesity is at risk for OSAS with daytime sleepiness, which is associated with the severity of liver damage independently of body mass and other cofactors.

## Introduction

Nonalcoholic fatty liver disease (NAFLD) is considered the hepatic manifestation of the metabolic syndrome related to systemic insulin resistance [1]. Paralleling the epidemics of obesity, NAFLD is becoming the most common chronic liver disease in Western countries, with a prevalence of 30% in the general population, which ramps up to 60–85% in at risk groups such as diabetics and obese [2]. NAFLD is characterized by a wide spectrum of histological liver damage, ranging from simple steatosis, usually a benign non-progressive condition, to nonalcoholic steatohepatitis (NASH) with possible evolution to significant fibrosis (stage >F1), which may evolve to clinically manifest liver disease, i.e. decompensated cirrhosis and hepatocellular carcinoma [3]. Although genetic variability is involved, factors explaining the susceptibility to progressive liver disease related to NAFLD are not completely understood [4], [5].

Obstructive sleep apnea syndrome (OSAS) is characterized by recurrent and intermittent episodes of hypoxia during sleeping due to the collapse of upper airways, resulting in fragmentation of sleep and excessive daytime somnolence [6]. OSAS is relatively frequent in the general population, but more so in obesity (prevalence 40%), which represents a major risk factor [7]. Therefore, OSAS clusters with metabolic syndrome manifestations, including NAFLD, and is a risk factor for diabetes [8]. OSAS contributes also to cardiovascular disease independently of classic metabolic alterations by inducing transient oxygen desaturation and adrenergic activation [9].

Evidence supports a role of oxidative stress in the progression from simple steatosis to progressive NASH [10], and hepatic ischemia-reperfusion injury related to OSAS may be implicated. Indeed, in severely obese NAFLD patients, intermittent hypoxia has been associated with insulin resistance and histological severity of liver damage [11], [12], [13]. These data are in line with a possible causal role of OSAS in the pathogenesis of liver damage in NAFLD, with potential therapeutic implications [7]. However, scant data are available on the relationship between OSAS and liver damage severity in patients with NAFLD without severe obesity [14], [15], [16], [17], who represent the majority of patients. This is particularly evident for the relationship between OSAS and histological features, which is the gold standard for prognostic assessment [5].

With this in mind, the aim of this study was to evaluate the relationship between the risk of OSAS, as evaluated by Berlin questionnaire (BQ) [18], the presence of excessive daytime sleepiness, assessed by Epworth sleepiness scale (ESS) [19], [20], [21], [22], and histological liver damage (presence of NASH and of significant fibrosis) in a series of Italian patients with NAFLD without morbid obesity.

## Materials and Methods

### Subjects

We evaluated 180 consecutive patients with clinical diagnosis of NAFLD aged between 18 and 70 years, with body mass index (BMI) lower than 35 Kg/m^2^, who underwent to liver biopsy between January 2009 and June 2013 at the Metabolic Liver Diseases service, Fondazione IRCCS Ca' Granda, for suspected NASH. Patients presenting with decompensated liver disease were excluded. OSAS risk was determined within 6 months from histological evaluation of liver damage. We excluded from the analyses patients for whom a weight change >2% was observed between liver biopsy and the compilation of questionnaires (n = 10), patients with other coexistent liver diseases [23] (n = 5), and those with an already established diagnosis of OSAS and/or under continuous positive airway pressure ventilation therapy (n = 6). Therefore, 159 were considered in the final analysis. Demographic and clinical features of patients are shown in table 1.

**Table 1:** Demographic and clinical features of 159 non-morbidly obese patients with histological NAFLD subdivided according to the presence of NASH.

	Overall series	Simple steatosis	NASH	p value
N =	159 (100)	83 (52)	76 (48)	
Gender F	39 (25)	15 (20)	24 (29)	0.20
Age years	50.8±12	50.8±12	50.7±12	0.98
BMI Kg/m^2^	27.4±3.9	26.3±3.4	28.4±4.1	0.008
Hypertension	54 (34)	27 (36)	27 (33)	0.73
Fasting glucose mg/dl	101±28	96±19	107±33	0.012
HOMA-IR	5.4±3.5	3.4±2.1	7.5±2.5	0.04
Total cholesterol mg/dl	202±41	205±38	198±45	0.26
HDL cholesterol mg/dl	48±13	49±13	46±13	0.25
Triglycerides mg/dl	143±76	134±76	151±76	0.14
Diabetes or IFG	46 (28)	17 (22)	29 (35)	0.11
Metabolic syndrome	63 (40)	23 (30)	40 (48)	0.02
ALT IU/ml	54±30	45±25	63±32	0.002
GGT IU/ml	82±95	76±89	88±100	0.45
Fibrosis stage >1	48 (30)	7 (9)	41 (49)	<0.0001

Data are shown as Mean ± SD (% values); according to data distribution; BMI: body mass index; IFG: impaired fasting glucose; HOMA-IR: homeostasis metabolic assessment insulin resistance index; ALT: alanine aminotransferases; GGT: gamma-glutamyl-transferase.

Controls (n = 80) were randomly selected from the 2554 participants in a population study carried out in the Milan area, whose cross-sectional phase has recently been completed (Phase 1 CAMELIA study, CArdiovascular risk, MEtabolic sindrome, LIver, and Autoimmune disease). The 1465 subjects with no ultrasonographic evidence of hepatic steatosis were matched 1∶2 with the study patients if they had the same age and gender, and were in the same body mass category (normal weight, 18.9–25 Kg/m^2^; overweight, 25–29.9 Kg/m^2^; obese, ≥30 Kg/m^2^). Twenty-one (26%) were women, mean age was 51±11 years, BMI 27.6±4.3 Kg/m^2^. As stipulated in the CAMELIA study protocol, all subjects underwent ultrasonographic evaluation of the liver with assessment of liver steatosis [24] and were administered the OSAS questionnaires at time of enrollment.

Biochemical data were measured by routine clinical tests. Insulin resistance was estimated by the Homeostasis Metabolic Assessment Insulin Resistance index (HOMA-IR) [25]. Diabetes and impaired fasting glucose were diagnosed according to World Health organization criteria [26], whereas metabolic syndrome was defined according to revised ATPIII criteria [27].

Written informed consent was obtained from each patient included in the study. The study was approved by the Ethical Committee of the Fondazione IRCCS Ca' Granda Milano and of Legnano Hospital (Milano), and it conforms to the principles of the Declaration of Helsinki.

### Liver Histology

Liver biopsies were performed via percutaneous technique. Assessment of steatosis, inflammation, ballooning, fibrosis and overall diagnosis of NASH was determined according to the NASH clinical research network criteria, and the severity of liver damage was graded and staged according to the NAFLD activity score (NAS) [28]. All biopsies are evaluated by a single expert pathologist (MM). The total NAS score represents the sum of scores for steatosis, lobular inflammation, and ballooning, and ranges from 0 to 8; whereas the hepatic fibrosis stage ranges from F0 to F4. Clinically significant fibrosis (i.e. at risk of progression towards clinically manifest liver disease) was defined as stage >F1 [29].

### Assessment of OSAS risk

Patients filled the two clinical questionnaires, BQ and ESS, in order to identify subjects at high risk for OSAS and the concomitant presence of daytime sleepiness, respectively, within 6 months from liver biopsy.

The BQ contains 10 questions covering three categories including: 1) snoring severity (items 1–5), 2) excessive daytime sleepiness (items 6–9), and 3) history of high blood pressure or obesity (item 10). The questionnaire also includes information about age, sex, height and weight. The patient is instructed to answer the questions in all three categories. The BQ was scored as previously reported by Netzer et al. [18]. If the individual scores are positive in at least 2 of the 3 categories, the patient is classified as being at high risk for OSAS, whereas if the patient scores positive in only one or none of the categories, then the patient is deemed to be at low risk for OSA.

The ESS is an eight-item questionnaire that is used to assess the presence of daytime sleepiness [19]. The questionnaire asks to respondents to rate from 0 to 3 their sleepiness in eight daily situations, giving a total score of 0 (no daytime sleepiness) to 24 (the most excessive daytime sleepiness). The cutoff point for excessive daytime sleepiness is considered to be equal or greater than 10 [20].

Therefore, we considered patients at high risk for OSAS those with positive BQ (BQ+), patients at high risk for OSAS and symptomatic for excessive daytime sleepiness (BQ+, ESS+) those who resulted positive for both BQ and ESS questionnaires.

### Statistical analysis

Variables are reported as mean and standard deviation (SD) or frequency, as appropriate. The χ^2^ test was used for categorical variables, and Student *t* test or ANOVA for continuous variables, when appropriate, to evaluate the statistical significances. The association between high risk of OSAS with or without excessive daytime sleepiness and the presence of NASH and histological liver damage was evaluated by multivariate logistic regression analysis analysis adjusted for biologically relevant confounders associated with NASH at univariate analysis. Differences were considered significant when p was <0.05 (two-tailed). Analyses were carried out by the JMP 10.0 Statistical analysis software (SAS, Cary, NJ, USA).

## Results

### Clinical features of study population

Clinical features of 159 non-morbidly obese patients with histological NAFLD are presented in table 1. They were mostly middle-aged (mean age 51 years) overweight (mean BMI 27.4) males (75%). Forty % had metabolic syndrome, and almost one third diabetes or IFG. Concerning liver damage, patients were almost equally distributed between NASH and simple steatosis. As expected (table 1), patients with NASH had higher insulin resistance, as indicated by higher BMI (p = 0.008), fasting glucose (p = 0.012), HOMA-IR (p = 0.04), and prevalence of metabolic syndrome (p = 0.02). Furthermore, they had higher ALT levels (p = 0.002) and prevalence of clinically significant hepatic fibrosis (stage >1; p<0.0001).

### Prevalence of positivity for OSAS risk scores

**Figure 1:**
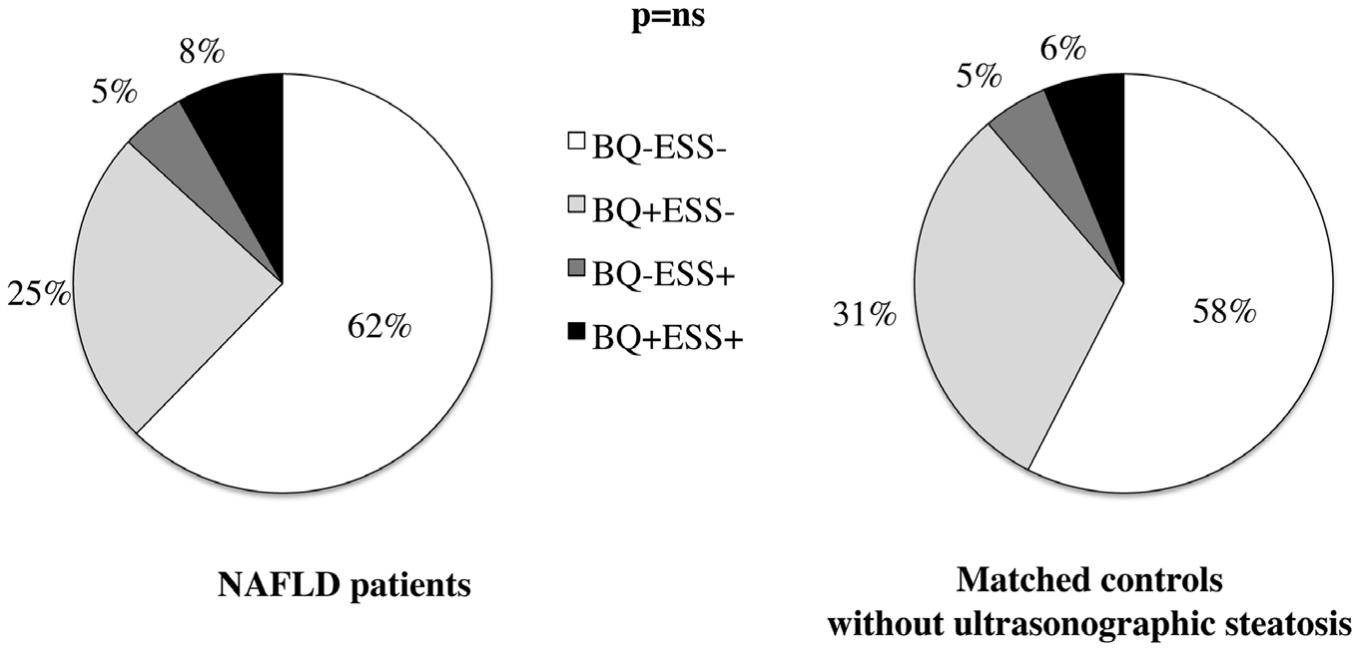
Prevalence of positivity for BQ alone (BQ+ESS−), ESS alone (BQ−ESS+), and both BQ and ESS (BQ+ESS+) in 159 non-morbidly obese patients with histological NAFLD and 80 matched controls without ultrasonographic evidence of hepatic steatosis. P = ns: not significant.

The prevalence of positivity for BQ and ESS in patients and controls without ultrasonographic steatosis is presented in figure 1. BQ and ESS were both positive in 13 patients (8%, BQ+ESS+, at high risk for OSAS and symptomatic for daytime sleepiness, henceforth OSAS with sleepiness), BQ alone was positive in 39 (25%, BQ+ESS−, at high risk for OSAS, but asymptomatic for daytime sleepiness), while ESS alone was positive in 8 (5%, BQ−ESS+, at low risk for OSAS, but symptomatic for hypersomnolence). Ninety nine patients (62%, BQ−ESS−) were negative for both questionnaires. Henceforth, we considered 52 BQ+ patients (33%) as being at high risk for OSAS, while the 13 (5%) BQ+ESS+ patients as at risk for OSAS with symptomatic daytime sleepiness.

The prevalence of positivity for OSAS risk scores in matched controls without ultrasonographic steatosis was superimposable to that of patients (46, 58% BQ−ESS−, 25, 31% BQ+ESS−, 4, 5% BQ−ESS+, 5, 6% BQ+ESS+; p = ns vs. patients; shown in figure 1).

### Effect of body mass on OSAS risk scores

Positivity for OSAS risk scores was influenced by body mass in patients (p<0.0001): in 37 mildly obese subjects 14 (38%) were BQ+ESS− and 9 (24%) BQ+ESS+, in 78 overweight individuals 18 (23%) were BQ+ESS− and 3 (4%) BQ+ESS+, among 44 normal weight patients 7 (16%) were BQ+ESS− and 1 (2%) BQ+ESS+.

The same trend was observed in matched controls without ultrasonographic steatosis (p = 0.005): in 19 obese subjects 12 (63%) were BQ+ESS− and 2 (10%) BQ+ESS+, in 39 overweight individuals 8 (20%) were BQ+ESS− and 3 (8%) BQ+ESS+, among 22 normal weight individuals 5 (23%) were BQ+ESS−, but none was BQ+ESS+ (p = ns vs. patients).

### Clinical features of patients at risk for OSAS with or without sleepiness

Clinical features of patients according to the risk of OSAS and the presence of daytime sleepiness are presented in table 2. No differences in gender distribution, age, insulin resistance and dyslipidemia were observed, but high risk for OSAS risk was associated with BMI, hypertension, and the metabolic syndrome (p<0.05). Serum levels of ALT tended to increase only in patients at risk for OSAS without sleepiness.

**Table 2:** Demographic and clinical features of 159 NAFLD patients according to the risk of OSAS with or without daytime sleepiness.

	High risk	High risk	Lower risk	P value
	Sleepiness	No sleepiness		
	(BQ+ ESS+)	(BQ+ ESS−)	(BQ−)	
N =	13 (8)	39 (25)	107 (67)	
Gender F	3 (23)	14 (36)	22 (21)	0.17
Age years	49.9±9.2	52.6±12.2	50.2±11.5	0.49
BMI Kg/m^2^	31.6±4.2*	28.9±4.3*	26.4±3.5	<0.0001
Hypertension	5 (38)	23 (59) *	26 (24)	0.0004
Fasting glucose mg/dl	99±13	104±29	101±29	0.73
HOMA-IR	6.0±3.9	4.0±2.1	5.9±2.8	0.74
Total cholesterol mg/dl	200±54	207±39	200±41	0.67
HDL cholesterol mg/dl	46±15	49±12	47±13	0.68
Triglycerides mg/dl	142±83	154±82	139±73	0.57
Diabetes or IFG	5 (38)	16 (41)	26 (24)	0.11
Metabolic syndrome	7 (54)	23 (59) *	33 (31)	0.005
ALT IU/ml	43±20	66±36*	51±28	0.01
GGT IU/ml	75±59	103±99	75±87	0.29

Data are shown as Mean ± SD (% values) according to data distribution; P value: at ANOVA; *P<0.05 vs. BQ− (t-test or Chi-square according to data distribution); BMI: body mass index; HOMA-IR: homeostasis metabolic assessment insulin resistance index; IFG: impaired fasting glucose; ALT: alanine aminotransferases; GGT: gamma-glutamyl-transferase.

### Association between OSAS risk and histological severity of liver damage

**Figure 2:**
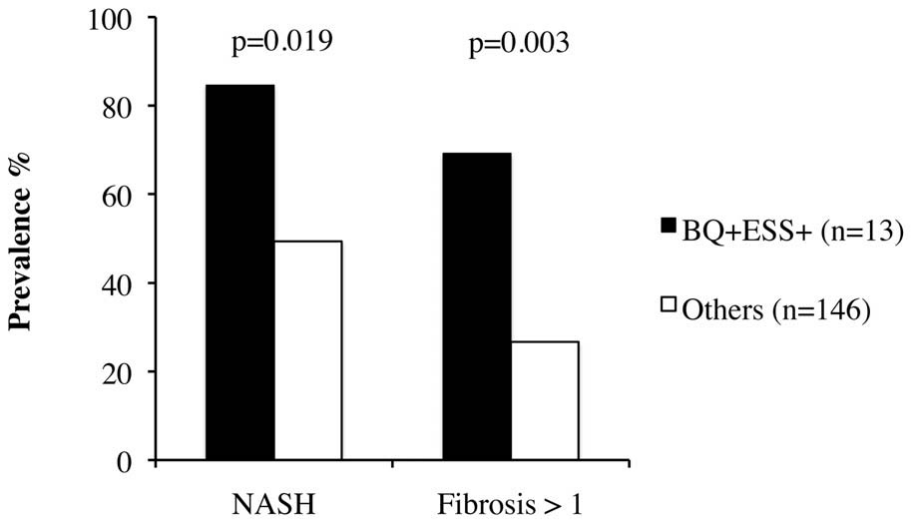
Association between high risk for OSAS with sleepiness (BQ+ESS+) vs. low risk (all other patients) with histological severity of liver disease (prevalence of NASH and of clinically significant fibrosis, i.e. stage >1) in 159 non-morbidly obese patients with histological NAFLD.

The relationship between risk of OSAS with sleepiness (high risk vs. low risk) and the severity of liver damage is shown in figure 2. Patients at risk of OSAS with sleepiness had higher prevalence of both NASH (p = 0.019) and fibrosis stage >1 (p = 0.003). Positivity for BQ alone (risk of OSAS without sleepiness) was not associated with increased risk of NASH (20/39, 51%, p = ns vs. BQ−ESS−) and fibrosis >1 (15/39, 38%, p = ns vs. BQ−ESS−).

**Figure 3:**
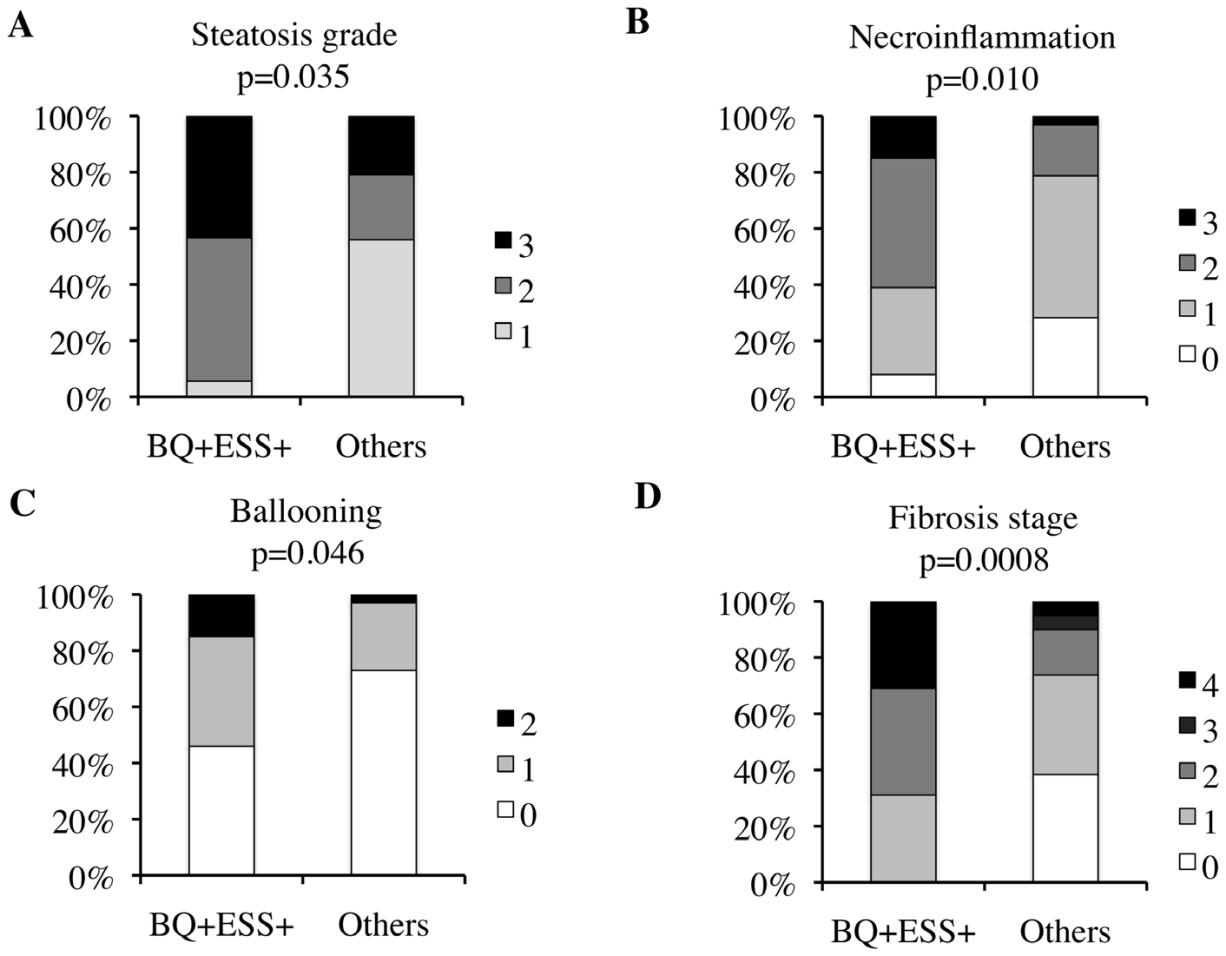
Association between high risk for OSAS with sleepiness (BQ+ESS+) vs. low risk (all other patients) with histological severity of liver disease as determined by components of NAFLD activity score (panel A: steatosis grade, panel B: necroinflammation, panel C: ballooning, panel D: fibrosis stage) in 159 non-morbidly obese patients with histological NAFLD.

The association of risk of OSAS with sleepiness with the severity of liver damage was evident for each component of the NAS score (shown in figure 3): steatosis grade (panel A, p = 0.049), necroinflammation (panel B, p = 0.010), hepatocellular ballooning (panel C, p = 0.046), and fibrosis stage (panel D, p = 0.0008).

We also performed a sensitivity analysis excluding patients with cirrhosis (n = 12, 8%), to evaluate the potential confounding effect of advanced liver disease on daytime hypersomnolence. High risk of OSAS with sleepiness remained associated with fibrosis stage >1 (5/9, 56% vs. 31/138, 22%; p = 0.04), nearly associated with NASH (7/9, 78% vs. 64/138, 46%; p = 0.06), and associated with steatosis grade (p = 0.04).

At multivariate logistic regression analysis (shown in table 3), high risk for OSAS with sleepiness was associated with both NASH (OR 7.13, 95% c.i 1.70–51.30) and fibrosis stage >1 (OR 14.03, 95% c.i. 3.45–70.11) independently of age, gender, hyperglycemia, BMI, and ALT levels. The presence of NASH was also independently associated with higher ALT levels, whereas fibrosis stage >1 with ALT levels and the presence of diabetes or IGT (table 3).

**Table 3:** Independent predictors of the severity of liver disease (presence of NASH and clinically significant fibrosis, i.e. stage >1) at multivariate logistic regression analysis in 159 patients with histological NAFLD.

	NASH			Fibrosis >1		
	OR	95% c.i.	p value	OR	95% c.i.	p value
Age years	0.98	0.95–1.01	0.37	0.98	0.94–1.01	0.28
Gender F	0.64	0.28–1.47	0.30	0.42	0.17–1.04	0.061
Diabetes or IFG	2.10	0.98–4.63	0.057	10.05	4.22–26.11	<0.0001
BMI Kg/m^2^	1.04	0.97–1.11	0.21	1.02	0.96–1.10	0.50
ALT IU/ml	1.02	1.01–1.03	0.008	1.02	1.01–1.03	0.004
High risk of OSAS with sleepiness (BQ+ESS+)	7.13	1.70–51.30	0.005	14.03	3.45–70.11	0.0002

OR: odds ratio; c.i.: confidence interval; high risk of OSAS with sleepiness: positivity for both BQ and ESS vs. all other patients. BMI: body mass index; IFG: impaired fasting glucose; ALT: alanine aminotransferases.

## Discussion

In this study, we evaluated the prevalence of high risk for OSAS with or without hypersomnolence, as determined by positivity for BQ and ESS, as compared to that of matched controls without ultrasonographic steatosis, and the association with histological liver damage in a series of non-morbidly obese patients with NAFLD. The study was prompted by previous evidence indicating that OSAS may be associated with the severity of NASH in morbidly obese patients [30], [31], [32], and in subjects with suspected OSAS [14], and that daytime somnolence has been associated with increased fatigue and autonomic dysfunction, possibly related to severe OSAS, in unselected NAFLD patients [33], [34].

We found that almost one in ten patients was at high risk for OSAS with sleepiness, whereas roughly another third of evaluated subjects was positive for BQ alone, being therefore at risk for OSAS, but asymptomatic for hypersomnolence. Futhermore, additional six patients were excluded from the study because of a previous confirmed diagnosis of OSAS. The prevalence of positivity for BQ was similar to that reported in a previous study in non-morbidly obese patients with NAFLD, which however did not evaluate ESS, which provides information on the presence of excessive daytime sleepiness [17]. As expected, the prevalence of OSAS and OSAS with somnolence increased with increasing body mass, but high risk of OSAS was also detected in some normal weight individuals.

However, the positivity for OSAS risk scores was not significantly higher to that of controls from a sample of the contemporary general population of the same geographical area, which was carefully matched for age, sex, and, most importantly, body mass. These data suggest that OSAS risk is linked with increased body mass, but the association is not influenced by fatty liver.

High risk for OSAS with sleepiness was associated with NASH and each component of the NAFLD score reflecting the activity of the disease, and it was even more strongly associated with the fibrosis severity, i.e. the stage of liver damage progression. In contrast, high risk for OSAS without symptomatic hypersomnolence was not associated with the severity of liver damage.

Importantly, even if risk of OSAS was linked with BMI, the association of risk for OSAS with sleepiness with liver damage was independent of BMI and of the other major clinical confounders, such as age, gender, the presence of hyperglycemia, and ALT levels. All in all, results suggest that the association between OSAS with somnolence and the severity of liver damage cannot simply be mediated by obesity or by the induction of insulin resistance and hyperglycemia.

The relationship between symptomatic high risk for OSAS and liver damage seemed also independent of advanced liver disease, as patients with decompensated cirrhosis were excluded from the study, and the association between high risk for OSAS with sleepiness and steatosis grade and fibrosis stage >1 was maintained even after exclusion of patients with compensated cirrhosis. Therefore, it seems unlikely that portal hypertension and hepatic dysfunction [35] account for the association between daytime somnolence and liver disease progression in the present study.

All in all, the present findings are consistent with the hypothesis that tissue hypoxia and reperfusion injury are involved in the pathogenesis of NASH and may contribute to fibrosis progression in a subset of NAFLD patients. In line with our data, in experimental models intermittent hepatic hypoxia favored lipogenesis, oxidative damage and inflammation, and activation of fibrogenesis [11], [12], [13].

Strengths of this study are represented by the systematic evaluation of non-morbidly obese patients with NAFLD, who still represent the majority of NAFLD cases in Italy and Europe [2], [36], and by the availability of liver histology in all patients, the gold standard for prognostic assessment [5]. Indeed, ALT levels and other noninvasive biomarkers, which were correlated with OSAS in previous studies as indices of liver damage [14], [15], [16] are insufficiently accurate biomarkers of the severity of liver disease [5], [37].

The main limitation of the study is represented by the lack of direct assessment of the presence and severity of OSAS by polysomnography, although BQ has been reported to have good accuracy in identifying patients at risk in the general population [18], [19], [20]. Furthermore, we cannot exclude that ultrasonography was not sensitive enough to rule out the presence of mild steatosis in some of high-risk control subjects.

Provided that the relationship between moderate to severe OSAS, liver damage, and fibrosis is confirmed by polysomonography in larger series of unselected patients with NAFLD, it would be worth assessing the effect of treatment with nocturnal continuous positive airway pressure ventilation on histological liver damage even in patients who would have otherwise borderline indication. Indeed, previous studies have suggested that nocturnal ventilation may improve markers of liver damage in patients with NAFLD and OSAS [38], [39].

## Conclusions

In conclusion, a proportion of NAFLD patients without morbid obesity, which is similar of that of matched subjects from the general population without ultrasonographic steatosis, is at high risk for OSAS with excessive daytime sleepiness, as detected by BQ and ESS combined positivity. High risk for OSAS with (but not without) sleepiness is independently associated with liver damage and fibrosis stage. Although the association between severe OSAS with sleepiness and the histological severity of NASH awaits confirmation by polysomnography, data suggest that OSAS may be involved in the pathogenesis of liver disease progression in NAFLD patients without morbid obesity, with potential therapeutic implications.
